# Electrochemical Properties of Tin Sulfide Nano-Sheets as Cathode Material for Lithium-Sulfur Batteries

**DOI:** 10.3389/fchem.2020.00254

**Published:** 2020-04-28

**Authors:** Muhammad Saleem, Gohar Mehboob, M. Shafiq Ahmed, Said Nasir Khisro, M. Z. Ansar, Kashif Mehmood, Rafiqa-Tul-Rasool, M. K. Alamgir, Ahsan Ejaz, M. Ghazanfar, Shahnwaz Hussain, Adil Ahmed, J. M. Ashfaq

**Affiliations:** ^1^Institute for Advanced Study, Shenzhen University, Shenzhen, China; ^2^Department of Optoelectronic Science and Technology, Shenzhen University, Shenzhen, China; ^3^Department of Physics, University of Kotli Azad Jammu and Kashmir, Kotli, Pakistan; ^4^National Institute of Vacuum Science and Technology, National Centre for Physics, Islamabad, Pakistan; ^5^Civil Engineering Department, Mirpur University of Science and Technology, Mirpur Azad Jammu and Kashmir, Mirpur, Pakistan; ^6^State Key Laboratory for Mechanical Behavior of Materials, School of Materials Science and Engineering, Xi'an Jiaotong University, Xi'an, China; ^7^School of Material Science and Engineering, Northwestern Polytechnical University, Shaanxi, China

**Keywords:** hydrothermal, nano-sheets, cathode material, tin sulfide, electrochemical impedance spectroscopy (EIS), cyclic voltammetry

## Abstract

Unprecedented self-assembled hierarchical nano-sheets of SnS were synthesized by the hydrothermal method. In a typical reaction, SnCl_2_.2H_2_O and Na_2_S.9H_2_O were used as reactants. Structural and morphological properties were studied by X-ray diffraction (XRD), and scanning electron microscopy (SEM) while the electrochemical properties were measured by cyclic voltammetry, charge-discharge cycles, and electrochemical impedance spectroscopy (EIS). SEM results showed the 1-D SnS nano-sheets with an average thickness of around 20 nm. Cyclic voltammogram and charge-discharge spectra showed good cycling stability. All these results showed that SnS nano-sheets are promising candidate material to be used as electrode for Li-S batteries.

## Introduction

The phenomenal progress in the development of a large number of electronic machinery is appearing to be a building headway in the advancement of up-to-a-minute secondary batteries. The demand for safer, environment friendly and more compact sized batteries for portable electronic devices is at a high. Metal chalcogenide semiconductor nano-materials are grabbing much more attention among building materials for electrodes of batteries. Li-S batteries is an auspicious contender for energy storage than that of common lithium-ion batteries and this is because of their high energy density (Manthiram et al., 2012). Li-S batteries are built from lithium anode and sulfur cathode with an electrolyte in between them. Li-S batteries possess a significant theoretical energy density i.e., 2,600 Wh/Kg and high theoretical specific capacity i.e., 1,675 mAh/g (Choi et al., 2012; Zhang, 2012). Their reported operating voltage is 2.1 V, which is compatible with devices which need a low operating voltage. These batteries deliver charge by the typical discharging process, in which S_8_ molecules undergo a reduction process by gaining electrons to produce lithium polysulfides (Li_2_S_*x*_). The discharging chain for Li-S batteries is given below:

(1) $$\begin{array}{l} {\text{S}}_{8}\to \text{L}{\text{i}}_{2}{\text{S}}_{8}\to \text{L}{\text{i}}_{2}{\text{S}}_{6}\to \text{L}{\text{i}}_{2}{\text{S}}_{4}\to \text{L}{\text{i}}_{2}{\text{S}}_{3}\to \text{L}{\text{i}}_{2}{\text{S}}_{2}\to \text{L}{\text{i}}_{2}\text{S} \end{array}$$

However, at the end of discharging process, Li_2_S is formed, which is insoluble in an electrolyte (Dominko et al., 2015). Among the major issues associated with Li-S batteries, which are insulating behavior of cathode, volume change, and charge storage efficiency (Fang and Peng, 2015; Ding et al., 2016; Pang et al., 2016), many attempts have been made to solve these issues e.g. nitrogen doped graphene oxide/sulfur nanocomposite used as electrode material by Qiu et al. (2014). Porous carbon/sulfur nanocomposite has been used as an electrode material by Jayaprakash et al. (2011). Mesoporous carbon nanoparticles has also used for Li-S batteries by Schuster et al. (2012). There were some improvements in the cyclic stability of the electrode material, however there was quiet low conductivity (0.0917E-24/cmΩ) of sulfur (Manthiram et al., 2012), operating efficiency, capacity and shuttling (Courtney and Dahn, 1997; Zhang, 2012), and these issues still remain to be fixed. The low electrical conductivity of sulfur, hinders the conduction of charge that restricts its direct use as cathode materials. To address the issue of low conductivity of sulfur, we suggest an alternate material for cathode material in this work.

SnS is a favorable candidate for Li-S batteries because of their high electronic conductivity with 9.17 × 10^4^/cmΩ and a high specific capacity of 994 mAh/g (Courtney et al., 1999; Armand and Tarascon, 2008). This high electronic conductivity suggests its use as cathode material instead of Li_2_S to solve the issue of insulating behavior of Li_2_S. Shujaat et al. (2017) used the hydrothermal method to synthesize SnS nanorods, however they have not used it for electrochemical study. Chaudhary et al. (2007) have synthesized SnS nanoflakes and studied its thermal properties. Tang et al. (2019) synthesized SnS polycrystals and studied its thermoelectric properties. Their methods and results helped immensely in understanding the properties of SnS. However, reports regarding SnS for its use as cathode material in Li-S batteries are still lacking. This material (SnS) is used as cathode to replace sulfur in Li-S batteries because it has good electrical conductivity as compared to sulfur which is insulating. Initially the performance is low, but it can be tuned in the future. In present research, tin-sulfide nano-sheets were successfully synthesized by the hydrothermal method. The prepared material was characterized by different techniques to check its suitability for its use as cathode material in batteries.

## Experimental Setup

### Fabrication of SnS Nano-Sheets

In the present study, hydrothermal method was used to prepare SnS nanoparticles by using tin chloride (SnCl_2_.2H_2_O) and sodium sulfide (Na_2_S.9H_2_O) as precursors. In a particular reaction, tin chloride and sodium sulfide provide Sn^2+^ and S^2^- ions respectively. 0.007 moles SnCl_2_.2H_2_O and 0.1 moles Na_2_S.9H_2_O were dissolved in 50 ml of ethylene glycol (EG) separately. Stoichiometric ratio of SnCl_2_.2H_2_O and Na_2_S.9H_2_O was 1:6 and after obtaining the fine solutions, SnCl_2_.2H_2_O was added very slowly in Na_2_S.9H_2_O solution using a pipette along with continuous magnetic stirring. During the mixing of two solutions, the mixture changed its color from light brown to blackish brown. The final solution was further treated hydrothermally by transferring it into a teflon-lined stainless steel autoclave and heated in oven at 200°C for 24 h. After 24 h, the autoclave was slowly cooled down to room temperature. A grey solid precipitate was obtained which was washed for eight times with DI water and ethanol to remove impurities and then dried at 70°C for 12 h. The formation of SnS nanoparticles in this work is based on the following double-displacement reaction under the basic conditions.

(2) $$\begin{array}{l} \text{SnC}{\text{l}}_{2}+\text{N}{\text{a}}_{2}\text{S}\to \text{SnS}+2\text{NaCl} \end{array}$$

The final grey powder was then annealed at 300°C for 1 h in order to remove defects and impurities, and saved for further characterizations.

### Preparation of Cathode Based on SnS

**Figure 1:**
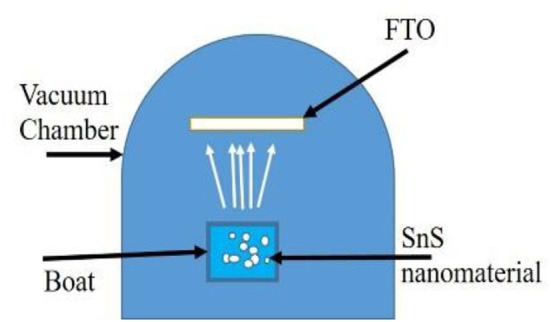
Schematic of thermal evaporation in a RHU used to deposite SnS thin film on FTO.

SnS thin film was deposited on FTO (fluorine doped tin oxide) coated in a glass substrate of a size of 1 cm^2^ by thermal evaporation in a resistive heating unit (RHU). Schematic of RHU is shown in the Figure 1 below.

The cleaned FTO substrate was placed in a holder of RHU. 0.04 gm, prepared material (SnS) was loaded in a tantalum boat of a heating unit and the chamber was evacuated till the vacuum level reached to 10^−5^ mbar. The boat was heated by applying a high voltage. SnS evaporated and deposited on the substrate. A good thin film with uniform thickness was deposited on the substrate and after cooling, the substrates were saved for characterizations.

The prepared SnS electrode material was characterized by using the different techniques. To check the crystallinity of the material, XRD was taken by a Bruker D_8_ ADVANCE X-ray diffractometer (XRD) having Cu-Kα rad (λ = 1.54056 Å). Morphological and elemental characterizations of SnS powder were done by using SEM (MERA3 TESCAN) and energy dispersive X-ray spectroscopy (EDX). To check the stability of the material during charging and discharging, cyclic voltammetry was performed. The EIS was performed by using GAMRY Reference 3000 potentiostate/galvanostate with three electrode system: (i) a working electrode that is glass/FTO/SnS, (ii) a counter electrode of graphite, and (iii) saturated calomel electrode (SCE) as a reference electrode. All electrochemical analysis have been taken in background electrolyte of LiClO_4_ salt in propylene carbonate (PC).

## Results and Discussion

### Structural and Compositional Characterization

The crystal structure of the prepared material was investigated by XRD.

**Figure 2:**
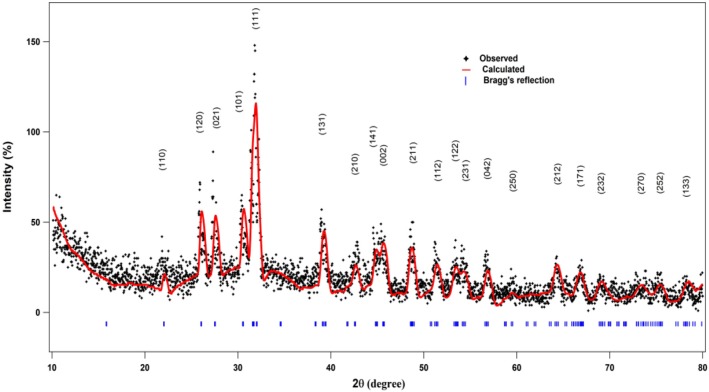
XRD pattern of SnS nanoparticles.

Figure 2 shows the XRD pattern of synthesized SnS nano-sheets. It can be seen that all the diffraction peaks correspond to orthorhombic crystal structure of SnS (herzenbergite crystal system) with lattice parameters *a* = 0.4329 nm, *b* = 1.1192 nm, *c* = 0.3984 nm and α = β = γ = 90° with space group number = 62 (JCPDS Cards no. 039-0354), confirming the formation of SnS material. No residual peaks are present in the XRD pattern which indicates the purity of the sample. The maximum intensity of an XRD peak at 2θ = 31.532 degree shows the crystal growth is in the direction of (111) plane. Strong and sharp peaks indicate the fine crystallinity of the prepared samples.

**Figure 3:**
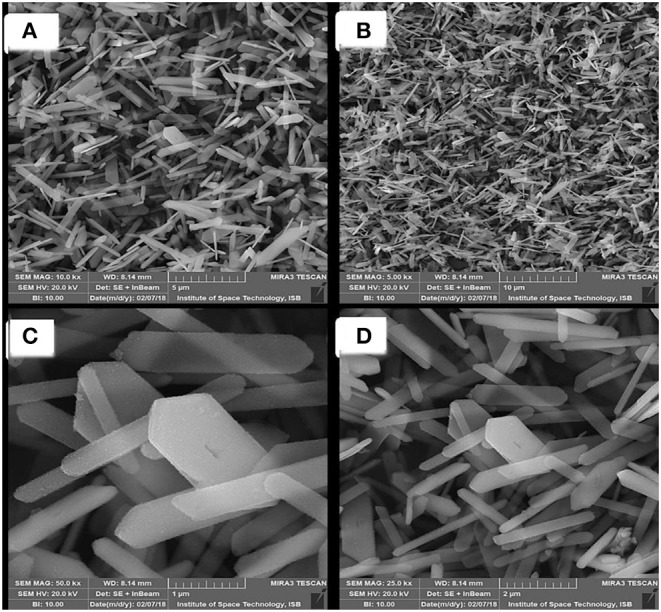
**(A,B)** low magnification and **(C,D)** high magnification images of prepared SnS nanosheets.

The morphology of the prepared SnS nanoparticles showed the 1-D fine rod like structures. The micrographs with low magnification shows the dendrites-like structures as shown in Figures 3A,B, while the high magnification micrographs reveals rod like structures as shown in Figures 3C,D. The selected scale of SEM was 1–10 μm, according to this scale the average size of prepared SnS nano-sheets is almost 200 nm which is calculated by SEM images.

**Figure 4:**
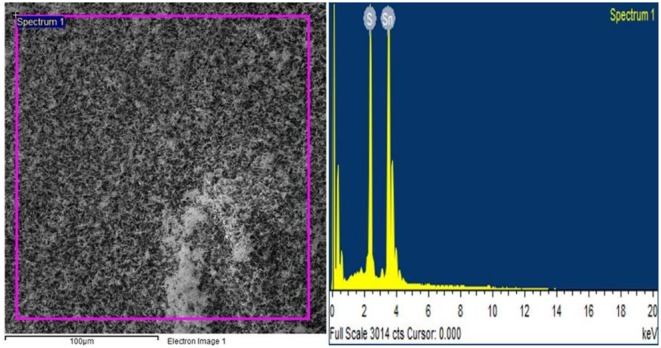
Elemental composition shown by EDX spectrum.

Elemental analysis of the specific area of the sample was conducted by using EDX which confirmed that there is no any other element present except S and Sn as shown in Figure 4.

### Electrochemical Characterization

**Figure 5:**
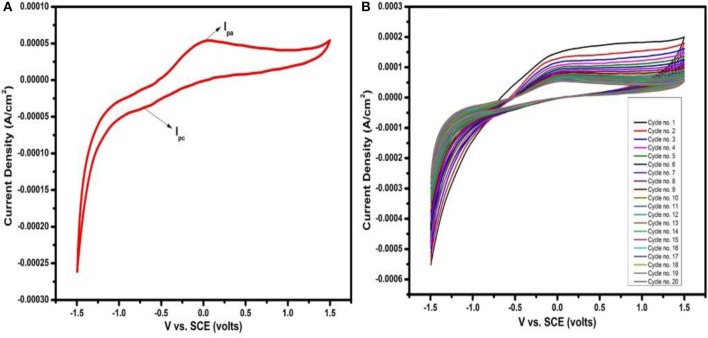
**(A)** Cyclic voltammetry glass/FTO/SnS electrode at scan rate of 100 mV/s and **(B)** shows 20 cycles at scan rate of 100 mV/s.

The cycling voltammetry was performed to characterize the oxidation and reduction reactions during electrochemical redox process of the deposited SnS based cathode. Cyclic voltammetry was performed in an electrolyte containing 0.5 molar lithium perchlorate (LiClO_4_) and 0.1 molar lithium nitrate (LiNO_3_) in propylene carbonate. Lithium nitrate was added to reduce shuttle mechanism during the electrochemical characterization. Cyclic voltammogram obtained at scan rate of 100 mV/s within a potential window from −1.5 to 1.5 V are shown in Figure 5A. The cyclic voltammogram possess a prominent anodic current peak (I_pa_) at 0.36 V and a cathodic current peak (I_pc_) at −0.69 V. The I_pa_ to the oxidation while I_pc_ refer to the reduction of SnS by Li^+^ ions as given by Equation (3);

(3) $$\begin{array}{l} 2\text{Li}+\text{SnS}↔\text{L}{\text{i}}_{2}\text{S}+\text{Sn} \end{array}$$

For cycling stability of the prepared material, 20 cycles of cyclic voltammetry were performed at a scan rate of 100 mV/s in a potential window of −1.5 to +1.5 V. It can be seen in Figure 5B that the SnS layer deposited on FTO used as cathode shows good cycling stability. The results show that this electrode material has good cycle reversibility and is much better than previous sulfur electrode (Jin et al., 2003).

**Figure 6:**
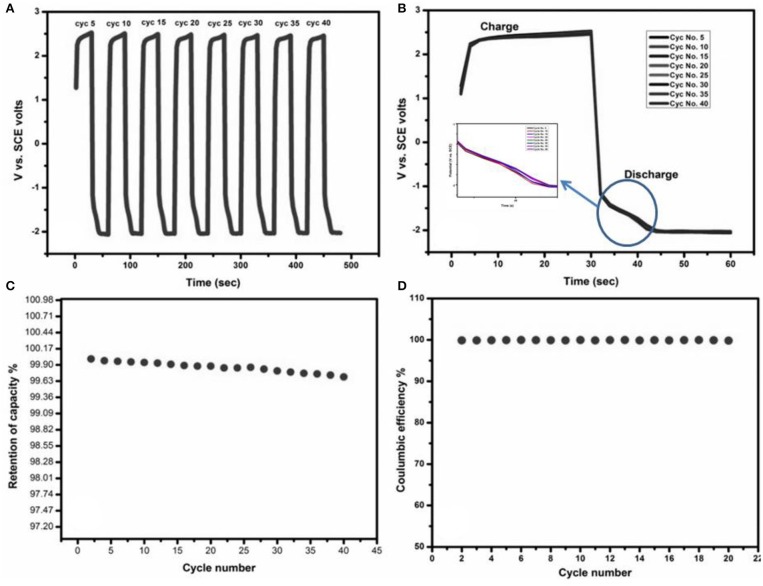
**(A,B)** Charge-discharge cycles upto 40 cycles, **(C)** percentage retention of capacity vs. cycle number, and **(D)** percentage coulumbic efficiency vs. cycle number. Inset elaborates multiple charge-discharge Cycles.

Charge/discharge behavior of the SnS layer deposited on FTO, used as electrode material was investigated for numerous cycles and the results are shown in Figure 6. It can be seen in Figures 6A,B that the SnS layer deposited on FTO has good stability up to 40 cycles during redox reaction. In the zoomed view in Figure 6B, it can be seen that after the charge region, there is the plateau region in discharge curve starting from −1.5 V, which indicates that the redox reactions occur within this region. In order to investigate the charge retention capacity of the material, the results of galvanostatic charge/discharge are shown in Figure 6C. In Figure 6, the percentage retention of capacity vs. cycle number and the capacity of SnS was retained at almost 99% even after 40 cycles. The coulombic efficiency of prepared electrode is shown in Figure 6D which is 100%, even after 20 cycles.

EIS is a beneficial experimental and means to characterize the frequency response of a device. The material that exhibits low resistances can be used for energy storage devices. Electrochemical parameters of the SnS electrode material are summarized in Table 1.

**Table 1:** Electrochemical parameters of the SnS electrode material.

**No**.	**Parameters**	**Cycle 1**	**Cycle 40**
1	Charge storage	578.1 μC	104.2 μC
2	Specific discharge capacity	8.4 μAh	8.4 μAh
3	Capacity retention	100%	99%
4	Columbic efficiency	100%	100%
5	ESR	75 ohms	

**Figure 7:**
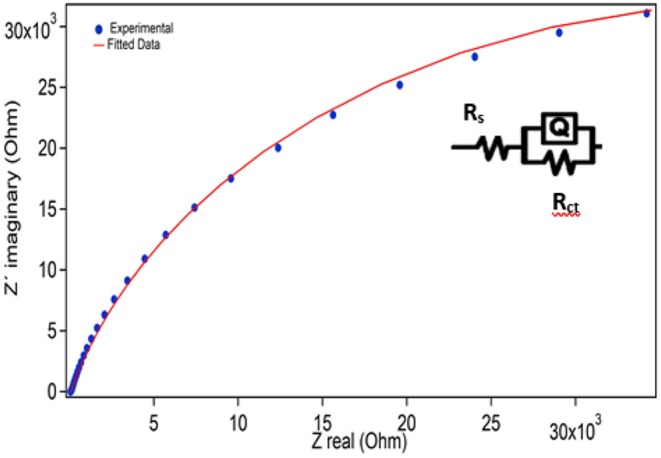
EIS spectrum for SnS electrode, inset shows the magnified view of lower value of Z_real_ and Z_img_.

EIS measurements were performed in LiClO_4_ and dissolved in propylene carbonate from 1 MHz to 0.1 Hz. Experimental data, extracted from the above-mentioned conditions, is normally plotted in a Nyquist plot which represents the imaginary part of the impedance vs. the real part. Figure 7 shows EIS spectra of SnS layer deposited on FTO.

The EIS data were fitted and analyzed by using the ZView software as shown in Figure 7. The inset demonstrates the equivalent circuit used to fit the impedance data, in which first resistance is in series with two electrode elements. In this equivalent circuit, R_s_, Q, and R_ct_ represent solution resistance, phase constant and charge resistance, respectively. The charge transfer resistance indicates that the deposition of SnS on FTO in LiClO_4_ + PC, is responsible for the control of the diffusional resistance. The results presented in Figure 7 show that SnS deposited on FTO offers an ESR of 75 Ω. ESR can be contribution of many parameters such as: electronic resistance, ionic resistance etc.

## Conclusions

SnS nano-sheets were successfully synthesized by template free hydrothermal method at the temperature of 200°C. XRD results confirmed the orthorhombic crystal structure of SnS material. 1-D fine nano-sheets were clearly seen in SEM micrographs and EDX results showed that no any other element present in the sample except Sn and S. Cyclic voltammogram performed at the scan rate of 100 mV/s, in different potential windows, showed the peaks of oxidation and reduction of material. The twenty cycles of cyclic voltammogram and forty charge-discharge cycles showed good cycling stability of electrode material prepared by depositing SnS thin film on FTO electrode. The plateau region of the discharge curve showed the occurrence of redox reactions of the material. EIS, through Nyquist plots showed that the equivalent series resistance of SnS is mainly diffusional control resistance. These results suggest that SnS nano-sheets are promising candidate to be used as a cathode material for lithium-sulfur batteries.

## Data Availability Statement

The datasets generated for this study are available on request to the corresponding author.

## Author Contributions

JA and MS design the experiment. GM conducted the experiments. Remaining authors (MSA, SK, MZA, KM, R-T-R, MKA, AE, MG, SH, and AA) contributed in different characterisation, experimental help, manuscript writing, proofreading, etc.

## Conflict of Interest

The authors declare that the research was conducted in the absence of any commercial or financial relationships that could be construed as a potential conflict of interest.
